# Ethical Issues Surrounding End-of-Life Care: A Narrative Review

**DOI:** 10.3390/healthcare4020024

**Published:** 2016-05-05

**Authors:** Sameera Karnik, Amar Kanekar

**Affiliations:** 1 American Public University, 111 West Congress Street, Charles Town, WV 25414, USA; sameera.karnik@gmail.com; 2 School of Counseling, Human Performance and Rehabilitation, University of Arkansas at Little Rock, 2801 South University Avenue, Little Rock, AR 72204, USA

**Keywords:** end-of-life care, ethics, treatments, advance directives

## Abstract

End-of-life care decision making carries paramount importance due to the advancements in medical sciences. Since medical science has evolved over the time and now has a potentiality to reshape the circumstances during death and in turn prolong lives, various ethical issues surround end-of-life care. The purpose of this narrative review is to discuss issues such as autonomous decision making, importance of advance directives, rationing of care in futile treatments and costs involved in providing end-of-life care. Even though much progress has been made in this area continued advancement in medical science demands further research into this topic.

## 1. Introduction

All human-beings are mortals and hence death is an inevitable occurrence. Advancements in medical technology are changing the norms of natural death. These technologically advanced treatments have a capability to intervene at the time of death and prolong the lives of people. Medical technologies are facilitating to reshape the circumstances around natural death, by sustaining human lives. Even though medical treatments have advanced technologically they hold no promises for recovery, they can sustain life with or without meaningful existence or with secondary support (like feeding tubes, ventilators, *etc.*). Hence, these medical advancements have empowered patients and their families (proxies) with an important task of choosing their treatment preference during end-of-life care [1].

“Decision-making” for end-of-life care has earned paramount importance as it has capability to prolong human life with the support of medical technologies or can let the natural death process continue by foregoing the treatment option [2]. Hence, end-of-life care is facing various ethical dilemmas. The purpose of this narrative review is to discuss issues such as autonomous decision making, importance of advance directives, rationing of care in futile treatments and costs involved in providing end-of-life care. This is a US centered study and the text does not necessarily apply to countries and contexts outside the United States.

## 2. Some Ethical Issues Surrounding End-of-Life Care

### 2.1. Autonomous Decision Making

“Decision making” is itself a very complex process of thoughts and sets-up various challenges for patients and their families to make up an end-of-life care decision [3]. Persons have a right to put forward their end-of-life treatment preferences. The Federal Patient Self-Determination Act (PSDA) effective since 1991 has facilitated communication between the healthcare providers and patients or consumers [4]. The person’s right to autonomously voice their end-of-life treatment choices has to be respected ethically considering the use of advance treatments and their prognosis. This right of autonomy has some limitations, and hence faces an ethical dilemma. The healthcare professional should respect the patient’s autonomy while considering its limitation and carry out their duties to benefit the patient without doing harm [5].

Even though we are discussing about patients right to autonomy we are talking about its limitations. To elaborate further, autonomy gives patients’ a right to control their treatment according to their preferences, though many a times their autonomy is not respected. They receive end-of-life care which is in-consistent with their end-of-life care preferences [6]. This gives importance to the ethical issue of autonomy surrounding end-of-life care preferences.

### 2.2. Physician’s Role and Responsibilities to Resolve the Issue

Healthcare professionals can play an important role by providing detailed information about an advanced medical treatment which can be used during end-of-life care. Physicians can perform their duties rightfully by providing patients detailed information about the benefits, limitations and drawbacks of that treatment. Physician can work according to “deontological theory” and perform their duties to gain greatest good for the patient and act for patients benefit [4]. Even though the patient has autonomy to choose a treatment, physician can explain its implications and try to emphasize on its consequences. Here, the patient has to perform a self-beneficence duty to take an autonomous decision as a competent individual to undergo the treatment and prolong life or forgo a futile treatment for the greatest good of society by saving cost and emotional stress. If the patient insists to prolong life with medically advanced treatment intervention, which according to physician evaluation might be futile, physician has the upmost responsibility to explain the information facts about withholding or withdrawing the medical treatment and see to it that there is no unnecessary utilization of resources for the futile treatment without causing harm to the patient. Physicians should respect the beliefs and values of that patient before withholding or withdrawing a treatment or giving an order for DNR (do not resuscitate) or resuscitation. Physician, additionally has a duty to preserve patient’s life but this duty is not to be confused with unnecessary use of resources and inflicting more harm than good to the patient by continuing medically futile treatments [5].

Physicians have to reach a mutual agreement with the patient about withholding or withdrawing a futile treatment and explain the drawbacks of unrealistic expectations from the treatment. Communication between patient and families, discussing patient’s goal regarding treatment and care, can be helpful to bridge a gap between the patient, their families and the physician [6].

### 2.3. Advance Directives

An “advance directive” enables competent individuals to design and document their health care decision plan in advance in case of future disability or terminal illness. This advance directive can be of two types, instructional and proxy, which allow competent individuals to make their healthcare choices in advance or specify their wishes to their providers or families in case of future disability in carrying out end-of-life decision [4]. This can give the patient an “individual autonomy” so as to receive end-of-life care consistent with their preference. In case of a competent individual, he or she can convey or document their end-of-life treatment preferences autonomously to the authorities on admission or can take a consensus autonomous decision after getting end-of-life care information from the physician as discussed above.

On the other hand in case of incapacitated individuals, families play a central role as proxies or primary care givers. Families have a responsibility of putting forth the end-of-life care preference of the patient. Family members play the role of proxy due to the virtue of their relationship with the patient and may not be very good in guessing the patients preference for end-of-life decision making, in the case where explicit declaration of patients preferences is not clear. These proxies try to judge the medical situation as the patient may have evaluated but it is seen that they are not very good at taking end-of-life decisions for the patients.

Families playing the crucial role of surrogate or proxy are emotionally attached to the incapacitated patient and hence their moral interest (emotional, financial pressure, *etc.*) may be diversified in opting for a treatment or declining them [4]. The unstable advance preferences may not be authentic in some eventful situations. Hence, the medical situation may need renewed evaluation and decision making. To address these ethical and legal issues arising from advance directives there is a need to educate the general population about the legal requirements and rights of the patient to accept or refuse a recommended treatment and advance directive. The information about proper implications and use of advance directives can facilitate in understanding and addressing legal issues arising from planning ahead by the patient or by the primary care givers [4].

### 2.4. Rationing of Care and Futile Treatment

The technological advancements and innovations are reshaping the decisions and treatment preferences surrounding end-of-life care. These technologically advanced treatments have a capability to prolong the life of a patient rather than allowing the natural dying process. The end-of-life decisions to sustain life are considered on the basis of patient centered care, quality of life after these advance treatments and have to be weighed along with shared decision-making process [1]. These new medical treatments and technologies are increasing the number of people seeking long-term care. It is challenging to provide long-term advanced treatment and care to the population considering the increase in older population and assessing the projected increase in this population, especially when the baby boom (a cohort born between 1946–1964) reach old age by 2030 [4]. It is assumed that people will adapt to healthy lifestyles and thus, this will reduce disabilities, diseases and injuries. This advancement will lower death rate and increase people living longer and needing long term care in their later life [4].

People should understand that they are mortals and consider getting information and making plans for end-of-life care preferences [7]. The futile and expensive treatment at end-of-life situations are increasing the unaffordable cost of healthcare and promoting inequitable healthcare. The ethical value of patient autonomy and surrogate autonomy should be respected but weighed against the use of expensive treatment in futile case circumstances with current increase in healthcare costs. Hence, in case of futile treatments, families and patients can ethically consider the option for comfort care. The advanced technologies hold no promises for recovery. These treatments can also lead to few humiliating and undignified situations for the patients which can be emotionally burdensome. Healthcare rationing of end-of-life care in futile situations can be considered as greatest good for society but has to be weighed against the patient autonomy [8].

It is difficult for the general population seeking medical care to understand the concept of limited treatment in case of futile cases. The stewardship of limiting medical care is surrounded by ethical issues as the patients and their families do not understand the need to limit treatment in some cases where it is futile. Healthcare providers and physician are working towards this challenging task of making patients understand the need to refuse treatment as it may not benefit them and in some cases can cause harm.

“Bioethics” points this limiting treatment or refusing futile treatment option as “rationing of care” in cases where unanimous decision about refusing advance treatment is not made collaboratively by patient and their healthcare providers. There are no strict criteria to differentiate futile treatment; hence it has to be relied on expert judgment and case prognosis. Considering the aspect of access of quality care to the people who need them most, the rationing of care in futile situation can be justified. Rationing of care is present in the current healthcare system and can be justified as equitable justice if carried out ethically and equitably [9]. Medical resource allocation is often limited and hence has to be distributed equitably. There is a need for evaluating and assessing the medically advanced treatment so as to avoid any undue use of already limited resources. This can be achieved by good education, knowledge about advanced treatment implications and improved healthcare decision making from patients, their families and physicians [7].

### 2.5. Costs Involved in End-of-Life Care

The expenditure on healthcare is too much in relation with total number of people and outcome. United States is spending a lot of money on health care and the average dollar amount per person is also much higher. Having said that, the health care expenditure is increasing, and at the same time people are spending more on getting the care they need. The cost of producing health care services due to advancement and innovations in technology is increasing the expenditure involved in providing these healthcare advanced treatment services. These healthcare services should not only target lengthening the life of people but also improve the quality of life [10], especially when end-of-life decisions and the costs involved in it are concerned. Compassionate care is another option seeked by the patients while considering end-of-life care which can be at times less costly and a good preference when medicine is unable to restore patient’s health. The medical treatments are financially burdensome to some patients; hence easy accessibility to quality care at affordable cost can lessen the financial issue adherent to the end-of-life care considering the increase in the unaffordability of healthcare [9].

### 2.6. Ethical Theories Involved in End-of-Life Care

Healthcare providers and physicians have to consider patient’s perspective and preferences. They have to work against the egoistic theory by working for the good of the patient [4]. Patient’s family members when implied with the task of making appropriate treatment choices or end-of-life care choices for the incapacitated patient should put aside their self-interest and judge the situation and come to a decision in the patient’s best interest. This act of working towards achieving greatest good for the patient by family members and by the physician can be termed under “Virtue theory” of ethics.

Physicians have to judge the situation and provide appropriate treatment prognosis so that patients’ can make an autonomous choice of treatment preferences or patients’ family can make these choices for them and work towards act of beneficence for the patient. While carrying out this act of beneficence, the physician has to provide information about the treatment, especially in case of futile treatment so as to avoid any undue harm to the patient. In case of futile treatments, healthcare providers also have to consider the allocation of limited resources available to manage the case scenario so as to avoid inequity. Hence, healthcare providers also have to consider the aspect of equitable and distributive justice in cases where expensive treatment provided to the patient during end-of-life situation may be futile, and utilize lot of resources, leading to unequal distribution of limited medical and technological resources [4]. Additionally, they have to address the issues of unnecessary and unequal distribution of resources by withdrawing or withholding the futile treatment [5].

## 3. Policy Implications

The task of healthcare executives to manage ethical issues surrounding end-of-life care is challenging. Healthcare executives can address these ethical dilemmas ensuring certain policies to be followed during managing this task. They can guide the patients and their surrogates to make informed treatment preferences by providing them trustful information, appropriate prognosis and available options regarding the case specific treatment choices. They can assist the patient and their families to make a well judged end-of-life care decision and document their preferences. In case there is a disagreement between the healthcare provider and the patient or surrogate end-of-life care choices, then they can take appropriate steps by appointing an ethics committee to address this ethical or legal issue and document its proceedings. Healthcare executives can compile policies, so as to introduce, promote, and discuss the use of advanced directives as an admission procedure [1]. This can motivate the patients to make a living will (advanced directive) about their end-of-life care preferences which in due process can facilitate families to make appropriate decisions in case of incapacitated patients.

Healthcare organizations can work towards developing and implementing guidelines & policies for end-of-life care decision making, especially policies for withholding or withdrawing the treatment options so as to avoid the ethical dilemmas. There should be a proper disclosure mentioning the limitations of certain specific treatment options if there are any, so that the patients and families are well informed about their treatment options and make well-judged decisions. Healthcare executives can develop resources supporting palliative treatment care choices. They can additionally provide detailed information and knowledge about these palliative care options so as to facilitate patients and their families to make a competent end-of-life care preference. Healthcare organizations can provide effective support by appointing an interdisciplinary ethics committee and employee assistance facility available so as to address any ethical crisis [1]. A well-formed, consistent and integrated ethics committee can safeguard organization’s future by increasing patient satisfaction, increasing organizations productivity, avoiding unethical activities, restricting undue costs, and reducing the risk of lawsuits [11].

## 4. Conclusions and Future Implications

Healthcare providers should take an initiative and discuss patient’s goal for end-of-life care or palliative care, as their preferences can change from person to person. Some patients might target for cure or some for comfort care, hence this trustful communication can avoid the ethical crisis surrounding that topic. The stability of these health preference goals is another issue as it has a potentiality to change with illness. Hence, the health scenario in each specific case has to be renewably evaluated so as to opt for scenario-based preferences. Here, the role played by clinicians is important as they can promote communication, education and discussion related to end-of-life care preferences and their implications among the patient, and their families in order to facilitate improved decision making. Effective advance planning or advanced directives can assist in putting forth patient’s autonomous choices but flexibility in these advanced directives can be appraised as it can accommodate any inadvertent scenario-based preference change and evaluation.

Community standards can work well where the patient’s desire from the end-of-life treatment choices is not well demarcated [3]. It is crucial to have a public dialogue discussing the ethical issues and dilemmas surrounding end-of-life care. This open discussion can facilitate development and implementation of policies and guidelines safeguarding the interest of patients and healthcare organizations [1]. Much progress has been made to address the ethical issues surrounding end-of-life care situation, and with the continued advancement in medical science, and its leading role in our lives demands further research into this topic. As age advances so thus the illness in many cases, hence there is a need to research and implement recommendations to relive the stress faced by people during that critical time and optimize quality care to improve and ease end-of-life journey [3].
